# Symptom clusters associated with complementary and alternative medicine use by cancer patients: A cross-sectional study

**DOI:** 10.1371/journal.pone.0294641

**Published:** 2023-12-14

**Authors:** Matthias Huemer, Anna Pansi, Guenter Hofmann, Angelika Terbuch, Elisabeth Sciri, Rainer Lipp, Jasmin Alija Spiegelberg, Daniela Jahn-Kuch, Martin Pichler

**Affiliations:** 1 Division of Oncology, Department of Internal Medicine, Palliative Care Unit, Medical University of Graz, Graz, Austria; 2 Division of Oncology, Department of Internal Medicine, Medical University of Graz, Graz, Austria; Korea University, REPUBLIC OF KOREA

## Abstract

**Background:**

Complementary and alternative medicine (CAM) is a commonly used approach among cancer patients, with a reported prevalence of 14.8 to 73.1% depending on multiple factors. Even though a typical patient-reported reason for using CAM is symptom control, no conclusive evidence could be established for specific symptoms being a predictor for CAM use. Symptom clusters are a novel approach for assessing a multidimensional symptom burden, reflecting the impact of diseases on the patient’s quality of life and considering the tendency of symptoms to occur in groups.

**Material and methods:**

A single-center cross-sectional study on cancer patients during oncological or palliative care was conducted to identify socio-demographical and clinical characteristics, including symptom clusters of CAM users and non-users. Symptom clusters were defined using latent profile analysis, and multivariable analyses were performed to assess significant factors influencing CAM use.

**Results:**

Of 171 cancer patients in this study, 63.7% used CAM alongside oncological treatment or palliative care. The most common CAM therapies were biological and physical therapies, including homeopathy (30.3%), supplements (27.4%), herbs (26.6%), massage (24.8%), and acupuncture (22.0%). Four distinct symptom clusters were identified, of which the cluster drowsiness-depression-anxiety was associated with a 3.83-fold increased chance of using CAM compared to low-symptomatic cancer patients. Multivariate analysis did not show any additional significant predictors of socio-demographical factors.

**Conclusion:**

Using the concept of symptom clusters revealed a significant predictor for CAM use, suggesting to be a more conclusive method for assessing symptom burden in cancer patients. In addition, understanding why and how cancer patients turn to CAM can enhance the quality of multidisciplinary communication about its use.

## Introduction

The use of complementary and alternative medicine (CAM) is a common practice among cancer patients, with an average prevalence of 51% and a range of 14.8 to 73.1% depending on geographical factors and the definition of CAM [1–3]. Currently, the most widely used definition of complementary therapies defines any health intervention that is not fully implemented into a public health care system as CAM, resulting in varying incidences of CAM use among cancer patients depending on the country and used definition of CAM in previous studies [1].

Current data identifies young females of higher educational levels as the main CAM users [1, 2], while also highlighting that psychological, cultural, and spiritual factors influence cancer patients’ tendency to use CAM [4, 5]. Patients commonly report using CAM to control and ease disease and treatment-related symptoms, but when evaluated using patient-reported outcome measures, the presence of specific symptoms and their severity did not correlate with CAM use [6, 7]. However, conflicting results exist regarding depressive symptoms as a predictor for spiritual CAM modalities such as prayers and spiritual healing in breast cancer patients [8].

Cancer patients’ symptoms often appear in groups or symptom clusters [9]. Currently, various definitions of symptom clusters exist, primarily defining them as two to three or more symptoms related to each other and appearing together [9]. The most common symptoms in cancer patients are pain, fatigue, and anorexia [10]. Each of these can be significantly disabling and create a self-perpetuating cycle when appearing together, therefore enhancing the perceived symptom burden for the patient. In contrast to single symptoms, such clusters interfere strongly with the patient’s quality of life [11]. A higher symptom impact in the quality of life was a predictor of CAM use in a population of cancer survivors [7].

Discussing CAM use with all cancer patients is essential to explore their potential needs, guide them in their choice of CAM, and also identify risks during cancer treatment, such as interactions between herbal therapies and tumor-specific remedies, which can either enhance their toxic side effects or impair the effectiveness of cancer treatment [12, 13]. However, not all patients report their CAM use to their oncologist in the course of their disease trajectory, making it difficult to estimate the risks of a changing behavior of patients that did not report the use of CAM in the past [14]. Finding patient characteristics associated with a higher chance of using CAM could raise oncologists’ awareness to actively assess CAM use in relevant patient populations. Symptoms are commonly evaluated during clinical practice and could be an easy screening tool for patients needing directed guidance concerning CAM if new symptoms occur or worsen in their severity which may change the tendency to use CAM of cancer patients [15]. Therefore, we aimed to investigate the relationships between symptom clusters and CAM use in a cross-sectional study of cancer patients.

## Methods

### Questionnaire design

We designed a paper-based self-completion questionnaire to engage patients using CAM in addition to their oncological care. The questionnaire was divided into three sections, including demographical data and patterns of CAM use, attitude towards CAM and holistic health, and symptom burden.

Demographic questions were collected to describe the diversity of patients participating in the study and to find potential sociodemographic factors associated with CAM use. The section included questions about sex, age, living status, educational level, yearly income. Data about the histologically confirmed cancer diagnosis, presence of metastasis, and current tumor-specific treatment were collected from the patients’ medical record. Further, we included a list of 24 treatment modalities used in previous studies to assess the most prevalent therapies of CAM, including homeopathy, traditional Chinese medicine, ayurveda, anthroposophical medicine, yoga, hypnosis, herbs, medical teas, vitamin/mineral supplements, massages, osteopathy, chiropractic, acupuncture, acupressure, qi gong/tai chi, meditation, prayers, dietary change, spiritual healing, shamanism, reiki/healing touch, healing stones/crystals, mistletoe therapy, shiatsu, and kinesiology [2]. This list was complemented by additional questions about the source of information about personal reasons for using CAM, its perceived positive and negative effects, and if its use was discussed with the responsible oncologist.

The following two sections included validated questionnaires exploring the attitude towards CAM and holistic health and symptom burden. First, we used the Holistic Complementary and Alternative Medicine Questionnaire (HCAMQ) to measure the attitude toward CAM [16, 17]. This two-factored patient-reported outcome measurement tool includes eight items creating two subscales, the holistic health (HH) subscale (four items) and the CAM subscale (four items). The HH factor describes a mental model consistent with the desire to avoid iatrogenic effects of conventional medicine and the belief that various lifestyle factors affect health. The items of HH assess the believe in how lifestyle factors including mood, stress and conflicts affect health. Additionally, the CAM subscale assesses the general attitude towards complementary therapies including the lack of its scientific evidence, whether CAM might be dangerous, when CAM should be used, how CAM might work and for which health conditions it should be used [16]. A higher score on each subscale reflects a greater positive belief in HH and CAM with acceptable psychometric properties of a Cronbach’s alpha of 0.75 and 0.83, respectively [17]. To evaluate symptom burden, we used the Edmonton Symptom Assessment Scale (ESAS), a widely used tool for assessing individual symptoms and their severity in cancer patients [18]. It includes 12 individual symptoms including pain, fatigue, drowsiness, nausea, anorexia, breathlessness, depression, anxiety, well-being, sleep, constipation, and emesis, rated on a numerical scale of 0 to 10, with higher scores reflecting greater symptom severity. To receive the ESAS total score, we summarized the single symptom scores of all 12 symptoms giving it a range of 0 to 120 [19].

#### Study participants and survey delivery

Data were collected in the clinics of the Division of Oncology and its associated Palliative Care Unit at the Department of Internal Medicine, Medical University of Graz, Austria. After obtaining the written informed consent of each participant, we handed out the paper-based questionnaire, which the participants completed on their own without further assistance by members of the study staff during their stay at the clinic. Eligible patients had histologically confirmed cancer, were at least 18 years old and should be able to read and complete the provided questions. Patients were excluded if there was a significant language barrier or were physically or mentally not able to complete the questionnaire. The completed questionnaires were returned to the study staff on the same day of participation. This study was performed in line with the principles of the Declaration of Helsinki. The local ethics committee approved the study (Ethics Committee of the Medical University of Graz, Austria; document number 32–665 ex 19/20).

#### Statistical analysis

The proportion of CAM users in Austrian oncological patients was previously reported to be 27% [3]. Therefore, to draw a representative sample with a comparable incidence with a margin of error of 6%, the required sample size is 171 patients. The sample size was calculated using G*Power (Version 3.1.9.6, 2020) [20].

The survey data was manually entered into a spreadsheet in Microsoft Excel format (Microsoft Corporation, 2019, version 2306, 64 bit) [21, 22]. For further analysis and data visualization, we used the statistical program R (RStudio, 2020, version 4.0.3) [23]. Differences in socio-demographical data between CAM users and non-users were calculated using the Chi^2^-test or Fishers’s exact test for categorical and the Wilcoxon-rank-sum-test for continuous variables. Finally, we used logistic regression for multivariable analysis using symptom clusters and the previously described predictors for CAM use sex, age and education as covariates.

For modeling symptom clusters, we performed a latent profile analysis (LPA) using the tidyLPA package in R [24]. LPA, as a form of exploratory factor analysis, is used to discover latent groups within a study sample, based on a certain set of variables [24]. In our study, we used LPA to discover groups of patients with similar response patterns on the symptoms list of the ESAS. The advantage of LPA compared to other clustering algorithms is that the algorithm also provides the mean severity scores of each item per group allowing a clear allocation of patients to each of the found clusters. The algorithm divides the population into different groups of patients with a specific symptom cluster, which we used for further analysis. We based our choice of the best LPA model on the Akaike information criterion (AIC), Bayesian information criterion (BIC), entropy, and clinical interpretability to achieve a parsimonious solution for the number of clusters [11].

Because LPA does not allow variables of different lengths, we had to impute missing ESAS values to perform the analysis without list-wise deletion and loss of statistical power. We analyzed the pattern of missing ESAS values and found that 30 were incomplete, with participants reporting only one or more symptoms with scores >0. We treated the missing values among these 30 patients as structurally missing and deduced the unreported symptoms as “not present.” We chose this approach assuming that patients, which did not report a value to one of the symptoms on the ESAS, where also not experiencing this specific symptom. Hence, we replaced missing ESAS values with 0. All other variables with missing data were analyzed with the data available.

## Results

### Sociodemographic and clinical characteristics

Between October 2020 and March 2021, 199 patients were asked to participate in the survey, of which 171 (50.9% female) completed the questionnaire. Most patients were at the age of 51–65 (45.0%), married (59.6%), completed primary education and apprenticeship (36.3%), and had a yearly income of € 10,001 to 20,000 (26.3%). The most frequent cancer diagnoses were breast cancer (24.0%), cancer of the lower gastrointestinal tract (23.4%), and pancreobiliary area (19.3%). A high proportion of the total sample had metastasized disease (87.7%) and received palliative tumor-specific treatment (67.8%). The sample had a moderate symptom burden of a mean ESAS total score of 27.2 (standard deviation [SD] 16.6). Detailed characteristics are listed in Table 1.

**Table 1:** Patient demographics and characteristics for the total sample, CAM users, and CAM non-users.

Patient demographics and characteristics	Total (n = 171)		CAM users (n = 109)		CAM non-users (n = 62)	
	N	Percent	N	Percent	N	Percent
**Age (years)**						
*18–36*	5	2.9	4	3.7	1	1.6
*37–50*	20	11.7	13	11.9	7	11.3
*51–65*	77	45.0	47	43.1	30	48.4
*66–80*	64	37.4	41	37.6	23	37.1
*>80*	5	2.9	4	3.7	1	1.6
**Sex**						
*Male*	84	49.1	58	53.2	26	41.9
*Female*	87	50.9	51	46.8	36	58.1
*Other*	0	0.0	0	0.0	0	0.0
**Civil status**						
*Single*	14	8.2	9	8.3	5	8.1
*Married*	102	59.6	70	64.2	32	51.6
*Divorced*	15	8.8	9	8.3	6	9.7
*Widowed*	7	4.1	6	5.5	1	1.6
*Missing*	33	19.3	15	13.8	18	29.0
**Education**						
*None*	3	1.8	2	1.8	1	1.6
*Primary*	12	7.0	6	5.5	6	9.7
*Apprenticeship*	62	36.3	35	32.1	27	43.5
*Higher secondary*	28	16.4	22	20.2	6	9.7
*Technical college*	29	17.0	17	15.6	12	19.4
*University*	28	16.4	20	18.3	8	12.9
*Other*	7	4.1	6	5.5	1	1.6
*Missing*	2	1.2	1	0.9	1	1.6
**Income per year**						
*<10 000 €*	21	12.3	15	13.8	6	9.7
*10 001–20 000 €*	45	26.3	27	24.8	18	29.0
*20 001–30 000 €*	34	19.9	24	22.0	10	16.1
*30 001–40 000 €*	30	17.5	16	14.7	14	22.6
*>40 000 €*	23	13.5	16	14.7	7	11.3
*Missing*	18	10.5	11	10.1	7	11.3
**Diagnosis**						
*Breast*	41	24.0	30	27.5	11	17.7
*Gynecological*	4	2.3	3	2.8	1	1.6
*Lung*	6	3.5	2	1.8	4	6.5
*Pancreobiliary*	33	19.3	22	20.2	11	17.7
*Upper gastrointestinal*	12	7.0	8	7.3	4	6.5
*Lower gastrointestinal*	40	23.4	23	21.1	17	27.4
*Urological*	12	7.0	8	7.3	4	6.5
*Prostate*	9	5.3	7	6.4	2	3.2
*CUP*	2	1.2	0	0.0	2	3.2
*Other*	12	7.0	6	5.5	6	9.7
**Metastasis**						
*None*	21	12.3	14	12.8	7	11.3
*Yes*	150	87.7	95	87.2	55	88.7
**Therapy**						
*Neo-adjuvant*	24	14.0	19	17.4	5	8.1
*Adjuvant*	19	11.1	12	11.0	7	11.3
*Palliative*	116	67.8	72	66.1	44	71.0
*Observation*	5	2.9	3	2.8	2	3.2
*Best supportive care*	7	4.1	3	2.8	4	6.5
**Immunotherapy**						
*Yes*	21	12.3	12	11.0	9	14.5
*None*	150	87.7	97	89.0	53	85.5
**ESAS**	Mean	SD	Mean	SD	Mean	SD
*Total score (0–120)*	27.2	16.6	28.3	16.5	25.5	16.9
**HCAMQ**						
*HH subscale*	21.3	2.7	21.4	2.4	21.1	3.0
*CAM subscale*	11.8	4.4	12.2	4.4	10.8	4.3

SD = standard deviation

### Use of complementary and alternative medicine

An unexpectedly high proportion of 63.7% within our sample used CAM during their cancer treatment. Detailed socio- and clinical demographics of CAM users and non-users are provided in Table 1. However, only 42.2% of CAM users discussed the topic with their oncologist before this survey, while 19.3% did not answer this question. The most common CAM modalities used were homeopathy (30.3%), vitamin/mineral supplementation (28.4%), herbs (26.6%), massage (24.8%), acupuncture (22.0%), and medical teas (22.0%). Most participants used CAM to strengthen their body (47.7%) and reported a subjective improvement in cancer treatment side effects (34.9%). Side effects related to CAM therapy were reported by 15.6%, with nausea being the most commonly addressed adverse reaction. Patients mostly got their information about CAM from family members (37.6%) and friends (45.0%). Detailed information on reasons for CAM use, perceived effects, and source of CAM are listed in Table 2.

**Table 2:** Reasons, perceived effects, and source of complementary and alternative medicine (CAM).

**CAM use**	Total (n = 171)	Percent
*Yes*	109	63.7
*No*	62	36.3
	Total (n = 109)	Percent
**Informed oncologist**		
*Yes*	46	42.2
*No*	42	38.5
*Missing*	21	19.3
**Reasons for CAM use (multiple choice)**		
*Fight cancer*	26	23.9
*Strengthen body*	52	47.7
*Symptom control*	20	18.3
*Enhance Quality of Life*	50	45.9
*Enhance emotional well-being*	30	27.5
*Enhance treatment effect*	31	28.4
*Lower treatment side effects*	37	33.9
*Take selfcontrol*	24	22.0
*Try everything*	34	31.2
**Subjective effects of CAM (multiple choice)**		
*Total reports*	83	76.1
*Reduced pain*	20	18.3
*Stress reduction*	15	13.8
*Reduced fatigue*	16	14.7
*Improved physical well-being*	37	33.9
*Improved emotional well-being*	24	22.0
*Improved Quality of Life*	28	25.7
*Improved immune system*	22	20.2
*Improved hope*	20	18.3
*fewer treatment side effects*	38	34.9
**Subjective side effects of CAM (multiple choice)**		
*Total reports*	17	15.6
*Abdominal pain*	4	3.7
*Nausea*	5	4.6
*Emesis*	1	0.9
*Headache*	1	0.9
*Diarrhea*	1	0.9
*Constipation*	0	0.0
*other*	8	7.3
**Source of information (multiple choice)**		
*Family*	41	37.6
*Friends*	49	45.0
*Internet*	18	16.5
*Other media*	20	18.3
*Family physician*	23	21.1
*Psychologist*	4	3.7
*Oncologist*	10	9.2
*Radiologist*	1	0.9
*CAM practitioner*	13	11.9
*Religious contact*	1	0.9
*Other*	11	10.1

### Symptom cluster analysis

To achieve an optimal symptom cluster solution, we performed exploratory LPA on increasing numbers of clusters and compared their model fit indices until the AIC and BIC plateaued (Table 3). This resulted in 1 to 8 possible clusters for further analysis. The model with the lowest BIC had 5 clusters. However, this solution would create two clusters that are very similar in terms of included symptoms and their severity. Because these would be hard to distinguish in a clinical setting, we selected the solution with 4 clusters to achieve a parsimonious model with balanced fit indices and clinical interpretability. Our model also showed a sufficient entropy greater than 0.8, indicating a correct group assignment of individual cases [25].

**Table 3:** Model fit indices of latent profile analysis for an increasing number of clusters.

Cluster	AIC	BIC	Entropy
1	9293.89	9369.29	1.00
2	8907.35	9023.59	0.89
3	8830.37	8987.45	0.82
4	8718.56	8916.48	0.87
5	8661.76	8900.53	0.87
6	8644.38	8923.99	0.80
7	8643.38	8963.83	0.90
8	8653.54	9014.83	0.91

**Fig 1:**
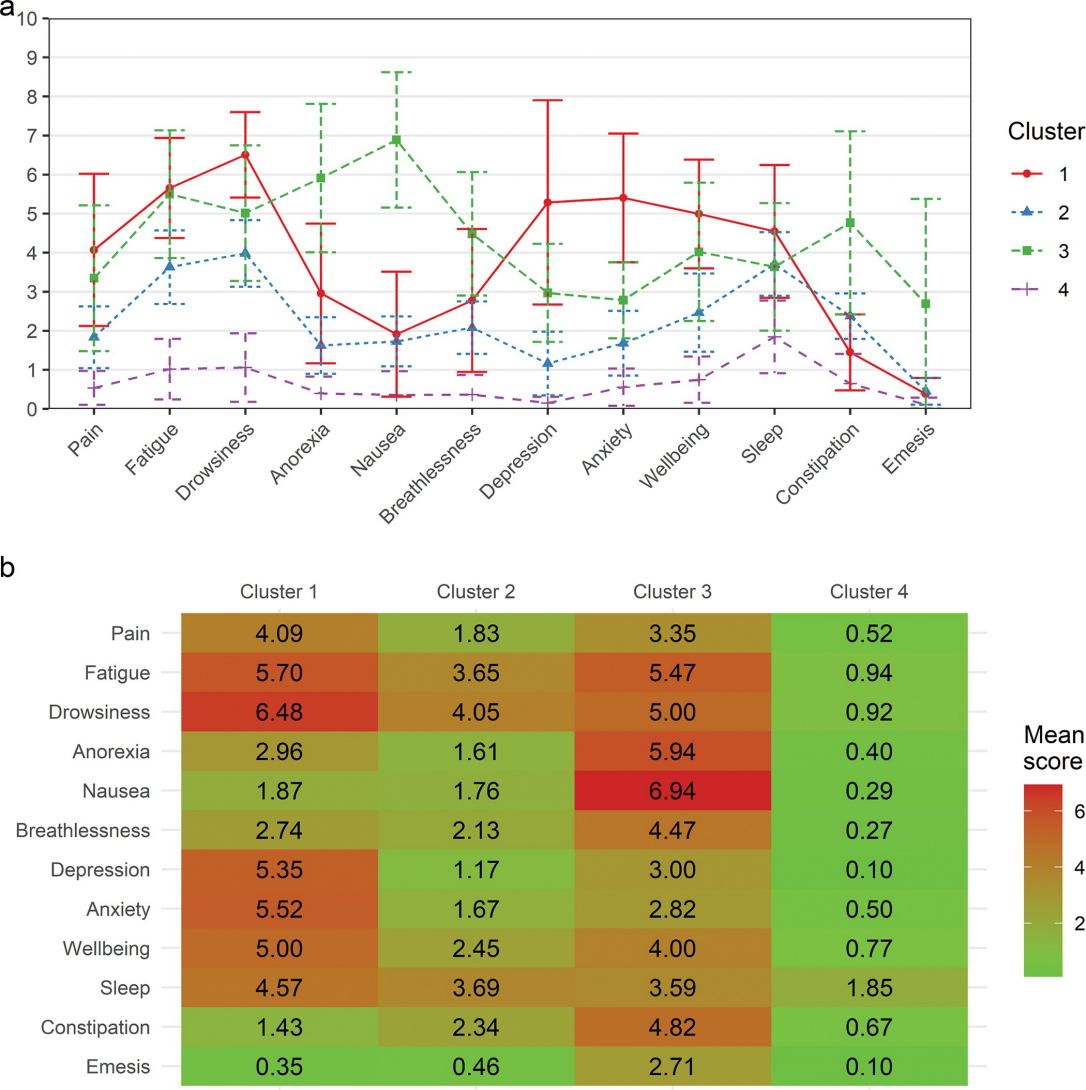
Symptom clusters resulting from latent profile analysis using a four-class solution. **a** Cluster 1: drowsiness-depression-anxiety-cluster, Cluster 2: moderate fatigue-drowsiness-sleep-cluster, Cluster 3: anorexia-nausea-breathlessness-constipation-emesis-cluster, Cluster 4: all-low-cluster; **b** Mean symptom scores of each cluster.

Our model yielded four clearly distinguishable symptom clusters (Fig 1). Clusters 2 and 4 are characterized by moderate fatigue-drowsiness-sleep and all-low scores, respectively. The remaining clusters 1 and 3 distinguish between drowsiness-depression-anxiety and anorexia-nausea-constipation-emesis scores, respectively, while both clusters had comparable high fatigue scores.

### Bivariate analysis

For using CAM, we found strong and statistically significant (p<0.05) bivariate associations for cluster membership (χ^2^ = 10.07, df = 3, p = 0.018) but no significant differences in symptom burden between CAM users and non-users (ESAS total score CAM 28.3±16.5 vs. non-CAM 25.5±16.9, p = 0.23). Further, both groups reported comparable symptom prevalence (CAM median 8 symptoms, interquartile range [IQR] 6, 10 vs. non-CAM median 7 symptoms, IQR 4.25, 9, p = 0.13). The attitudes towards CAM and HH did also not differ significantly between CAM users and non-users (CAM subscale 12.2±4.4 vs. 11.0±4.5, p = 0.08; HH subscale 21.4±2.4 vs. 21.1±3.2, p = 0.65).

Interestingly, we could not identify any bivariate associations between CAM use and socio-demographical characteristics, including the previously described predictors [1] sex (χ^2^ = 1.58, df = 1, p = 0.208), age (p = 0.909 Fisher’s exact test), and education (p = 0.259 Fisher’s exact test). Additionally, no other variables showed significant associations with the outcome variable (Table 4).

**Table 4:** Results of bivariate analysis for socio-demographics.

Patient demographics and characteristics	CAM users (n = 109)		CAM non-users (n = 62)		P-value
	N	Percent	N	Percent	
**Age (years)**					0.909 Fisher’s exact
*18–36*	4	3.7	1	1.6	
*37–50*	13	11.9	7	11.3	
*51–65*	47	43.1	30	48.4	
*66–80*	41	37.6	23	37.1	
*>80*	4	3.7	1	1.6	
**Sex**					χ^2^ = 1.58, df = 1, p = 0.208
*Male*	58	53.2	26	41.9	
*Female*	51	46.8	36	58.1	
*Other*	0	0.0	0	0.0	
**Civil status**					p = 0.699 Fisher’s exct
*Single*	9	8.3	5	8.1	
*Married*	70	64.2	32	51.6	
*Divorced*	9	8.3	6	9.7	
*Widowed*	6	5.5	1	1.6	
*Missing*	15	13.8	18	29.0	
**Education**					p = 0.259 Fisher’s exact
*None*	2	1.8	1	1.6	
*Primary*	6	5.5	6	9.7	
*Apprenticeship*	35	32.1	27	43.5	
*Higher secondary*	22	20.2	6	9.7	
*Technical college*	17	15.6	12	19.4	
*University*	20	18.3	8	12.9	
*Other*	6	5.5	1	1.6	
*Missing*	1	0.9	1	1.6	
**Income per year**					χ^2^ = 3.25, df = 4, p = 0.517
*<10 000 €*	15	13.8	6	9.7	
*10 001–20 000 €*	27	24.8	18	29.0	
*20 001–30 000 €*	24	22.0	10	16.1	
*30 001–40 000 €*	16	14.7	14	22.6	
*>40 000 €*	16	14.7	7	11.3	
*Missing*	11	10.1	7	11.3	
**Diagnosis**					p = 0.362 Fisher’s exact
*Breast*	30	27.5	11	17.7	
*Gynecological*	3	2.8	1	1.6	
*Lung*	2	1.8	4	6.5	
*Pancreobiliary*	22	20.2	11	17.7	
*Upper gastrointestinal*	8	7.3	4	6.5	
*Lower gastrointestinal*	23	21.1	17	27.4	
*Urological*	8	7.3	4	6.5	
*Prostate*	7	6.4	2	3.2	
*CUP*	0	0.0	2	3.2	
*Other*	6	5.5	6	9.7	
**Metastasis**					χ^2^ = 0.003, df = 1, p = 0.956
*None*	14	12.8	7	11.3	
*Yes*	95	87.2	55	88.7	
**Therapy**					p = 0.396 Fisher’s exact
*Neo-adjuvant*	19	17.4	5	8.1	
*Adjuvant*	12	11.0	7	11.3	
*Palliative*	72	66.1	44	71.0	
*Observation*	3	2.8	2	3.2	
*Best supportive care*	3	2.8	4	6.5	
**Immunotherapy**					χ^2^ = 0.18, df = 1, p = 0.668
*Yes*	12	11.0	9	14.5	
*None*	97	89.0	53	85.5	

## Multivariable analysis

We fitted a multiple logistic regression model using symptom cluster membership and the previously described factors of sex, age, and education as predictors for CAM use. Table 5 shows the fully adjusted model.

**Table 5:** Results of multivariable analysis.

Predictor	CAM use (yes = 1)		
	OR	95% CI	p-value
**Sex (Ref. = male)**			
*Female*	1.52	0.76, 3.09	0.242
**Symptom-cluster (Ref. = 4)**			
*1 = drowsiness-depression-anxiety*	3.83	1.14, 15.48	0.039
*2 = moderate fatigue-drowsiness-sleep*	2.09	0.96, 4.59	0.062
*3 = anorexia-nausea-breathlessness-constipation-emesis*	0.57	0.17, 1.88	0.362
**Age (Ref. = >80 years)**			
*18–36*	0.44	0.01, 14.99	0.613
*37–50*	0.23	0.01, 2.24	0.251
*51–65*	0.26	0.01, 1.77	0.209
*66–80*	0.25	0.01, 2.02	0.250
**Education (Ref. = none)**			
*Primary*	0.81	0.03, 12.93	0.881
*Apprenticeship*	0.98	0.04, 12.62	0.989
*Higher Secondary*	2.77	0.11, 40.63	0.464
*Technical college*	0.99	0.04, 13.74	0.999
*University*	1.81	0.07, 25.22	0.667
*Other*	6.30	0.16, 266.26	0.290

Ref. = reference

Cluster 1 (drowsiness-depression-anxiety-cluster) was the only significant predictor of all included variables for CAM use. Patients being part of this cluster had a 3.83-fold higher chance in using CAM (95% confidence interval [CI] 1.14, 15.48; p = 0.039) compared to cluster 4 (all-low-cluster). In contrast, Cluster 3 (anorexia-nausea-breathlessness-constipation-emesis-cluster) showed a 0.57-fold decreased chance of using CAM, although not significant. Female sex, lower age, and higher education showed a trend towards a higher chance of using CAM. However, these predictors did not reach statistical significance in our model.

## Discussion

CAM is a highly relevant topic in cancer patients as it can have positive as well as harmful effects on the oncological treatment outcome. However, factors influencing its use are insufficiently understood. It should be noted that the circumstances increasing the chance of patients using CAM are undoubtedly multifactorial, encompassing bio-psycho-sociocultural factors. Our study identified a new factor contributing to a higher willingness to use CAM during oncological treatment.

Despite previous findings, our multivariable analysis showed a robust statistical association between a multidimensional symptom burden and the use of CAM. We found the chance of using CAM to be 3.83-fold higher for the symptom-cluster drowsiness-depression-anxiety compared to low-symptomatic cancer patients. This is in contrast to previous studies, finding no associations to single symptoms or summative scores like the ESAS total score [6, 7], but complements the findings of Wu et al. showing a higher chance for CAM use by symptom interference with the quality of life of patients [7].

Further, we could identify the relevant symptoms contributing to the higher willingness to use CAM. Cluster 1 and cluster 3 have comparable high symptom scores. However, compared to cluster 1, patients with cluster 3 membership showed an insignificant trend toward a decreased chance of using CAM. This could be explained by several factors, including better symptom management or the persistence of the cluster symptoms. Compared to depressive and exhausting symptoms, gastrointestinal symptoms like nausea (the most prominent symptom in cluster 3) may be temporary, self-limiting, or better manageable with pharmaceutical interventions. Fatigue and depressive symptoms are among the most distressing for cancer patients with limited treatment options, possibly explaining the higher prevalence of CAM use in our sample [26]. This is also reflected by the two most reported reasons for using CAM among all users: the wish to strengthen the body (47.7%) and the wish to improve quality of life (45.9%). In contrast, 33.9% of participants reported ‘to lower treatment side effects,’ and only 18.3% stated controlling their symptoms with complementary therapies. Symptoms associated to cancer and tumor-specific therapies change throughout the disease trajectory, may worsen in severity and impact the patient’s quality of life as the disease progresses. While the use of CAM should be discussed with every cancer patient as early as possible, a significant change in the symptom burden of patients such as the appearance of the drowsiness-depression-anxiety cluster may serve as an indicator for oncologists to reassess the use of CAM in this patient population. Schofield et al. published a guide for effectively discussing CAM in an oncology setting based on a systematic review [15]. In this review, the authors suggest to actively assess CAM use at critical points during a patient’s disease trajectory including the commencement of a new treatment regime and new occurring symptoms [15].

Other studies suggested using CAM as an active problem-solving coping behavior or as a result of dissatisfaction with their treating physician rather than psychological distress per se [27, 28]. Unmet needs in symptom management may urge certain patients to seek help from ‘outside’ the boundaries of ‘conventional medicine’ as far as omitting cancer treatment in favor of CAM. This may also explain the higher risk of death of CAM users because of treatment refusal [13]. The high prevalence of CAM in our sample and in numerous other countries might reflect the need for professional symptom management and an integrative approach compromising rehabilitative, early palliative, supportive care, and evidence-based complementary medicine during oncological treatment [2, 8].

Guiding oncology patients in terms of CAM, including open discussions about its use during oncological treatment, is of utmost importance to prevent interactions or even treatment refusal. Our sample mainly used safe CAM modalities, including physical therapies like massage and acupuncture [29]. However, the reported and prevalent therapies also included biological-based therapies like herbs and medical teas, prone to interactions with tumor-specific treatments [30]. Most patients get their information about CAM from their close social environment (friends 45.0%, family 37.6%) and by media (internet 16.5%, other media 18.3%), while substantial low proportions received guidance from medical trained professions (family physician 21.1%, CAM practitioner 11.9%, oncologist 9.2%, radiologist 0.9%). Additionally, only 42.2% of all CAM users discussed their CAM use with an oncologist. The nonnegligible 19.2% of denied answers to this question suggest a greater communication gap, as this item might be subject to a social desirability bias, and the outcome might be underreported. Keeping the skewness of the information-seeking behavior towards unprofessional sources in mind, risks associated with CAM might also result from this communication gap between physicians and patients. On the other hand, enhancing open communication about CAM can strengthen the physician-patient relationship and lead to a greater satisfaction with cancer care in general [2, 14]. The use of CAM should therefore be part of routine history taking, particularly at important changes within the disease trajectory. When discussing the topic of CAM, it is important to explain relevant concerns from a scientific point of view while considering and respecting the cultural diversity and epistemological believe systems of patients [15]. In some cases, it may also be useful to guide patients in their choice of CAM towards treatment modalities which are to some extend evidence-based and safe [2, 15]. Recently published clinical guidelines by leading oncological associations offer an overview of different CAM therapies, their possible benefits and harms in an oncology setting for various symptoms [31–36].

Our study was conducted during the COVID-19 pandemic, which led to a higher degree of uncertainty for cancer patients concerning the availability of tumor-specific treatment, the risk of COVID-19 infection, and its potential interference with the treatment plan. The resulting stress associated with the pandemic enhanced the perceived symptom burden and increased the incidence of anxiety and depression [37–40]. Additionally, the pandemic created a new barrier to access health care services for cancer patients causing a delayed start of tumorspecific treatment and reduced access to symptom management [41, 42]. This may have also changed the information seeking behavior of the patients in our study, leading to a greater motivation to manage their occuring symptoms themselves and using other sources including the family members, friends and media including the internet, which is also reflected by our results. In our study, we found a higher proportion of cancer patients using CAM compared to a survey among Austrian cancer patients before the COVID-19 pandemic (63.7% versus 27.3%). Further, we could show that the chances of using CAM were associated with the symptom cluster that included mainly psychological symptoms [3]. Hence, the pandemic could have influenced the results of our study. However, several factors could explain the higher incidence of CAM use, including its increasing popularity over time, in general.

Our study has certain other limitations, up-front the single-center design limiting the generalizability of our findings in terms of cultural and socio-demographical factors. Our results may therefore be not applicable in areas in which traditional and complementary medicine represents a significant proportion of the local public health care system [43]. However, we did assess the attitude towards CAM using a validated measurement tool (HCAMQ). We found no statistically significant difference between the users and non-users of CAM in our sample, suggesting no significant influence of the individual cultural preoccupation. Additionally, we only included patients currently in oncological or palliative care at our clinic. Hence, our associations are only applicable to patients using CAM complementary to conventional medicine and not alternatively, meaning if they quit oncological treatment in favor of CAM, which could be a completely different patient cohort with different socio-demographical, clinical, and motivational characteristics. Nevertheless, the study’s strengths, including a prospective study design, robust sample size, a diverse sample, and validated patient-reported outcome measures, support our finding that symptom clusters are a significant and measurable predictor of CAM in oncology patients.

## Conclusion

For the first time, we could show the value of symptom burden using the concept of symptom clusters as a measurable predictor for CAM use in cancer patients. Our results indicate that patients with the symptom cluster “drowsiness-depression-anxiety” have a 3.83-fold higher chance to use CAM therapies than low-symptomatic patients, which is why we suggest to monitor the symptom burden of patients to detect significant changes of symptoms during the disease trajectory and reassess the use of CAM as early as possible in relevant patient populations. Further, we found the main source of information about CAM to be their family and friends and the media including the internet. This highlights the necessity to raise the awareness of clinical physicians to actively assess and discuss the use of CAM with their cancer patients.

## Supporting information

S1 Checklist STROBE statement—Checklist of items that should be included in reports of *cross-sectional studies*. (DOC)

## References

[1] Keene MR, Heslop IM, Sabesan SS, Glass BD. Complementary and alternative medicine use in cancer: A systematic review. Complement Ther Clin Pract. 2019;35:33–47. Epub 2019/04/21. doi: 10.1016/j.ctcp.2019.01.004 . 31003679

[2] Molassiotis A, Fernández-Ortega P, Pud D, Ozden G, Scott JA, Panteli V, et al. Use of complementary and alternative medicine in cancer patients: a European survey. Ann Oncol. 2005;16(4):655–63. Epub 2005/02/09. doi: 10.1093/annonc/mdi110 . 15699021

[3] Spiegel W, Zidek T, Vutuc C, Maier M, Isak K, Micksche M. Complementary therapies in cancer patients: prevalence and patients’ motives. Wien Klin Wochenschr. 2003;115(19–20):705–9. Epub 2003/12/04. doi: 10.1007/BF03040886 . 14650945

[4] Song S, Cohen AJ, Lui H, Mmonu NA, Brody H, Patino G, et al. Use of GoFundMe(®) to crowdfund complementary and alternative medicine treatments for cancer. J Cancer Res Clin Oncol. 2020;146(7):1857–65. Epub 2020/03/29. doi: 10.1007/s00432-020-03191-0 . 32219517 PMC11804722

[5] Verhoef MJ, Balneaves LG, Boon HS, Vroegindewey A. Reasons for and characteristics associated with complementary and alternative medicine use among adult cancer patients: a systematic review. Integr Cancer Ther. 2005;4(4):274–86. Epub 2005/11/12. doi: 10.1177/1534735405282361 . 16282504

[6] Chui PL, Abdullah KL, Wong LP, Taib NA. Complementary and Alternative Medicine Use and Symptom Burden in Women Undergoing Chemotherapy for Breast Cancer in Malaysia. Cancer Nursing. 2018;41(3):189–99. doi: 10.1097/NCC.0000000000000527 -201805000-00004. 28723722

[7] Wu H-J, Tai C-J, Tai C-J, Chien L-Y. Symptom severity, symptom interference and use of complementary and alternative medicine among survivors of colorectal and breast cancer after curative treatment in Taiwan. European Journal of Cancer Care. 2019;28(1):e12925. doi: 10.1111/ecc.12925 30276957

[8] Montazeri A, Sajadian A, Ebrahimi M, Akbari ME. Depression and the use of complementary medicine among breast cancer patients. Supportive Care in Cancer. 2005;13(5):339–42. doi: 10.1007/s00520-004-0709-z 15549425

[9] Kim H-J, McGuire DB, Tulman L, Barsevick AM. Symptom Clusters: Concept Analysis and Clinical Implications for Cancer Nursing. Cancer Nursing. 2005;28(4):270–82. doi: 10.1097/00002820-200507000-00005 -200507000-00005. 16046888

[10] Teunissen SCCM, Wesker W, Kruitwagen C, de Haes HCJM, Voest EE, de Graeff A. Symptom Prevalence in Patients with Incurable Cancer: A Systematic Review. Journal of Pain and Symptom Management. 2007;34(1):94–104. doi: 10.1016/j.jpainsymman.2006.10.015 17509812

[11] Kaufmann TL, Getz KD, Hsu JY, Bennett AV, Takvorian SU, Kamal AH, et al. Identification of Patient-Reported Outcome Phenotypes Among Oncology Patients With Palliative Care Needs. JCO Oncology Practice. 2021;17(10):e1473–e88. doi: 10.1200/OP.20.00849 . 33760637 PMC8791824

[12] Alsharif F. Discovering the Use of Complementary and Alternative Medicine in Oncology Patients: A Systematic Literature Review. Evid Based Complement Alternat Med. 2021;2021:6619243. Epub 2021/02/02. doi: 10.1155/2021/6619243 ; PubMed Central PMCID: PMC7817268. 33519943 PMC7817268

[13] Johnson SB, Park HS, Gross CP, Yu JB. Complementary Medicine, Refusal of Conventional Cancer Therapy, and Survival Among Patients With Curable Cancers. JAMA Oncology. 2018;4(10):1375–81. doi: 10.1001/jamaoncol.2018.2487 30027204 PMC6233773

[14] Davis EL, Oh B, Butow PN, Mullan BA, Clarke S. Cancer patient disclosure and patient-doctor communication of complementary and alternative medicine use: a systematic review. Oncologist. 2012;17(11):1475–81. Epub 2012/08/31. doi: 10.1634/theoncologist.2012-0223 ; PubMed Central PMCID: PMC3500370 22933591 PMC3500370

[15] Schofield P, Diggens J, Charleson C, Marigliani R, Jefford M. Effectively discussing complementary and alternative medicine in a conventional oncology setting: Communication recommendations for clinicians. Patient Education and Counseling. 2010;79(2):143–51. doi: 10.1016/j.pec.2009.07.038 19783116

[16] Hyland ME, Lewith GT, Westoby C. Developing a measure of attitudes: the holistic complementary and alternative medicine questionnaire. Complement Ther Med. 2003;11(1):33–8. Epub 2003/04/02. doi: 10.1016/s0965-2299(02)00113-9 . 12667973

[17] Kersten P, White PJ, Tennant A. Construct Validity of the Holistic Complementary and Alternative Medicines Questionnaire (HCAMQ)-An Investigation Using Modern Psychometric Approaches. Evid Based Complement Alternat Med. 2011;2011:396327. Epub 2009/10/02. doi: 10.1093/ecam/nep141 ; PubMed Central PMCID: PMC3135427. 19793835 PMC3135427

[18] Bruera E, Kuehn N, Miller MJ, Selmser P, Macmillan K. The Edmonton Symptom Assessment System (ESAS): a simple method for the assessment of palliative care patients. J Palliat Care. 1991;7(2):6–9. Epub 1991/01/01. . 1714502

[19] Chang VT, Hwang SS, Feuerman M. Validation of the Edmonton Symptom Assessment Scale. Cancer. 2000;88(9):2164–71. Epub 2000/05/17. doi: 10.1002/(sici)1097-0142(20000501)88:9&lt;2164::aid-cncr24&gt;3.0.co;2-5 . 10813730

[20] Faul F, Erdfelder E, Lang AG, Buchner A. G*Power 3: a flexible statistical power analysis program for the social, behavioral, and biomedical sciences. Behav Res Methods. 2007;39(2):175–91. Epub 2007/08/19. doi: 10.3758/bf03193146 . 17695343

[21] Corporation M. Microsoft Excel. 2019 (16.0) ed2018.

[22] Huemer M, Pichler M. Symptom clusters associated with complementary and alternative medicine use by cancer patients: A cross-sectional study. Figshare. 2023. doi: 10.6084/m9.figshare.23969121.v1 PMC10721086 38096236

[23] Team RC. R: A language and environment for statistical computing. R Foundation for Statistical Computing. Vienna, Austria 2020.

[24] Rosenberg J, Beymer P, Anderson D, Schmidt J. tidyLPA: An R Package to Easily Carry Out Latent Profile Analysis (LPA) Using Open-Source or Commercial Software. Journal of Open Source Software. 2018;3:978. doi: 10.21105/joss.00978

[25] Ramaswamy V, Desarbo WS, Reibstein DJ, Robinson WT. An Empirical Pooling Approach for Estimating Marketing Mix Elasticities with PIMS Data. Marketing Science. 1993;12(1):103–24.

[26] Dhingra L, Barrett M, Knotkova H, Chen J, Riggs A, Lee B, et al. Symptom Distress Among Diverse Patients Referred for Community-Based Palliative Care: Sociodemographic and Medical Correlates. Journal of Pain and Symptom Management. 2018;55(2):290–6. doi: 10.1016/j.jpainsymman.2017.08.015 28844624

[27] Montazeri A, Sajadian A, Ebrahimi M, Haghighat S, Harirchi I. Factors predicting the use of complementary and alternative therapies among cancer patients in Iran. European Journal of Cancer Care. 2007;16(2):144–9. doi: 10.1111/j.1365-2354.2006.00722.x 17371423

[28] Söllner W, Maislinger S, DeVries A, Steixner E, Rumpold G, Lukas P. Use of complementary and alternative medicine by cancer patients is not associated with perceived distress or poor compliance with standard treatment but with active coping behavior. Cancer. 2000;89(4):873–80. 10.1002/1097-0142(20000815)89:4<873::AID-CNCR21>3.0.CO;2-K. 10951352

[29] Birch S, Bovey M, Alraek T, Robinson N, Kim T-H, Lee MS. Acupuncture as a Treatment Within Integrative Health for Palliative Care: A Brief Narrative Review of Evidence and Recommendations. The Journal of Alternative and Complementary Medicine. 2020;26(9):786–93. doi: 10.1089/acm.2020.0032 32924554

[30] Wolf CPJG, Rachow T, Ernst T, Hochhaus A, Zomorodbakhsch B, Foller S, et al. Interactions in cancer treatment considering cancer therapy, concomitant medications, food, herbal medicine and other supplements. Journal of Cancer Research and Clinical Oncology. 2022;148(2):461–73. doi: 10.1007/s00432-021-03625-3 33864520 PMC8800918

[31] Leitlinienprogramm Onkologie (Deutsche Krebsgesellschaft DK, AWMF):. Komplementärmedizin in der Behandlung von onkologischen PatientInnen, Langversion 1.1: AWMF Registernummer: 032/055OL; 2021. Available from: https://www.leitlinienprogramm-onkologie.de/leitlinien/komplementaermedizin/.

[32] Lyman GH, Greenlee H, Bohlke K, Bao T, DeMichele AM, Deng GE, et al. Integrative Therapies During and After Breast Cancer Treatment: ASCO Endorsement of the SIO Clinical Practice Guideline. Journal of Clinical Oncology. 2018;36(25):2647–55. doi: 10.1200/JCO.2018.79.2721 . 29889605 PMC13123314

[33] Mao JJ, Ismaila N, Bao T, Barton D, Ben-Arye E, Garland EL, et al. Integrative Medicine for Pain Management in Oncology: Society for Integrative Oncology–ASCO Guideline. Journal of Clinical Oncology. 2022;40(34):3998–4024. doi: 10.1200/JCO.22.01357 . 36122322

[34] National Comprehensive Cancer Network. Cancer-Related Fatigue (Version 2.2023) 2023 [March 3, 2023]. Available from: https://www.nccn.org/professionals/physician_gls/pdf/fatigue.pdf.

[35] National Comprehensive Cancer Network. Antiemesis (Version 1.2023). 2023.

[36] Network NCC. Adult Cancer Pain (Version 2.2022). 2022.

[37] Ayubi E, Bashirian S, Khazaei S. Depression and Anxiety Among Patients with Cancer During COVID-19 Pandemic: A Systematic Review and Meta-analysis. J Gastrointest Cancer. 2021;52(2):499–507. Epub 2021/05/06. doi: 10.1007/s12029-021-00643-9 ; PubMed Central PMCID: PMC8096890. 33950368 PMC8096890

[38] Christine M, Steven MP, Karin S, Maura A, Hala B, Susan C, et al. Stress and Symptom Burden in Oncology Patients During the COVID-19 Pandemic. Journal of Pain and Symptom Management. 2020;60(5):e25–e34. MIASKOWSKI2020e25. doi: 10.1016/j.jpainsymman.2020.08.037 32889039 PMC7462969

[39] Miaskowski C, Paul SM, Snowberg K, Abbott M, Borno HT, Chang SM, et al. Loneliness and symptom burden in oncology patients during the COVID-19 pandemic. Cancer. 2021;127(17):3246–53. Epub 2021/04/28. doi: 10.1002/cncr.33603 . 33905528 PMC9508796

[40] Yildirim OA, Poyraz K, Erdur E. Depression and anxiety in cancer patients before and during the SARS-CoV-2 pandemic: association with treatment delays. Qual Life Res. 2021;30(7):1903–12. Epub 2021/02/27. doi: 10.1007/s11136-021-02795-4 ; PubMed Central PMCID: PMC7907665. 33635508 PMC7907665

[41] Ali JK, Riches JC. The Impact of the COVID-19 Pandemic on Oncology Care and Clinical Trials. Cancers [Internet]. 2021; 13(23). doi: 10.3390/cancers13235924 34885038 PMC8656780

[42] Pujolar G, Oliver-Anglès A, Vargas I, Vázquez M-L. Changes in Access to Health Services during the COVID-19 Pandemic: A Scoping Review. International Journal of Environmental Research and Public Health [Internet]. 2022; 19(3). doi: 10.3390/ijerph19031749 35162772 PMC8834942

[43] Organization WH. WHO traditional medicine strategy: 2014–2023: World Health Organization; 2013.

